# Physical activity profile of hypertensive adults living in rural South Africa

**DOI:** 10.4102/sajp.v81i1.2134

**Published:** 2025-04-23

**Authors:** Kganetso Sekome, Hellen Myezwa, F. Xavier Gómez-Olivé, Lauren B. Sherar, Dale Esliger

**Affiliations:** 1 Department of Physiotherapy, Faculty of Health Sciences, University of the Witwatersrand, Johannesburg, South Africa; 2 School of Sport, Exercise and Health Sciences, Loughborough University, Loughborough, United Kingdom; 3 Department of Therapeutic Sciences, Faculty of Health Sciences, University of the Witwatersrand, Johannesburg, South Africa; 4 MRC/Wits Rural Public Health and Health Transitions Research Unit, School of Public Health, Faculty of Health Sciences, University of the Witwatersrand, Johannesburg, South Africa

**Keywords:** IPAQ, hypertension, high blood pressure, sedentary, exercise

## Abstract

**Background:**

Understanding the physical activity behaviours of adults at risk of poor health is important to inform targeted interventions. We profiled the frequency, intensity, duration and domain (work or non-work) of self-reported physical activities of rural South African adults living with hypertension.

**Objectives:**

The aim of this study was to provide a profile of the frequency, intensity, duration and domain of self-reported physical activities over a 7-day period in adults with hypertension from a rural sub-district in South Africa.

**Method:**

A total of 429 adults diagnosed with hypertension aged 40 years and above completed the International Physical Activity Questionnaire Long-Form via telephone interview. Data were summarised using means and standard deviations or medians and interquartile ranges. The Mann–Whitney U test and Krustal–Wallis rank test were used to assess physical activity differences by sex and work status. Statistical significance was set at *p* < 0.05.

**Results:**

The mean age of the participants was 65.1 (standard deviation [s.d.] ± 10.9 years), 58% were women and 52% had paid or unpaid work. Men reported greater (duration and frequency) vigorous physical activity at work compared to women (*p* = 0.003 and *p* = 0.002). Walking frequency as a mode of transport was higher for working men and women (*p* < 0.001). Women reported higher frequency of moderate-intensity physical activity inside the house (*p* < 0.001) and outside the house (*p* < 0.001) compared to men. Non-working men and women spent more time sitting during the week compared to their working counterparts (*p* = 0.009).

**Conclusion:**

The physical activity profile of hypertensive adults varied by sex and work status.

**Clinical implications:**

Contextual factors such as gender roles are also related to the physical activity profile of hypertensive adults living in rural South Africa and should be considered when designing specific interventions targeted at improving hypertension control for this population.

## Introduction

Hypertension is a global public health concern that ranks first as a cause of disability-adjusted life years worldwide (Carey et al. 2018). The prevalence of hypertension is on the rise worldwide, especially in low- and middle-income countries, making hypertension a worldwide public health concern (Mills, Stefanescu & He 2020). Data from the World Health Organization show the national prevalence of hypertension in South Africa to be approximately 27.4% for men and 26.1% for women (World Health Organization & GHO, 2015), while a pooled prevalence of up to 60% has been previously reported (Gaziano et al. 2017). For rural areas of South Africa, the highest recently recorded prevalence of hypertension for adults was 52% (Abrahams-Gessel et al. 2023). Hypertension, defined as systolic blood pressure of ≥ 140 mmHg and diastolic blood pressure of ≥ 90 mmHg, is a modifiable risk factor for cardiovascular and cerebrovascular diseases such as heart attacks and strokes, which causes premature adult deaths in South Africa (Valenzuela et al. 2021). In comparison with urban areas, rural areas of South Africa have a higher prevalence of cardiovascular and cerebrovascular diseases (Akinyemi et al. 2021; Ntuli et al. 2015). This high prevalence may partly be attributed to the ageing population found mainly in rural areas (Kobayashi et al. 2021).

The high prevalence of hypertension against the backdrop of poorly organised health care systems, intermittent drug supply and shortage of health care workers (Rispel et al. 2019; Sekome 2018; Zihindulai, MacGregor, & Ross 2018) is a major concern, and consequently, the control of the condition is often left to the patient (Zihindulai et al. 2018). Most hypertensive patients in rural South Africa have focused their management of hypertension on anti-hypertensive medication, but this has shown little evidence of effectiveness (Abrahams-Gessel et al. 2023; Jardim et al. 2017; Kayima et al. 2013). Lifestyle approaches in combination with pharmaceutical therapy (Bertram et al. 2012; He et al. 2013; Lin et al. 2014) have been shown to provide optimal control of hypertension (Valenzuela et al. 2021). The widely recommended lifestyle approaches to control hypertension are a reduction in salt and caloric intake and an increase in physical activity (Bertram et al. 2012; He et al. 2013; Lin et al. 2014).

The positive effects of physical activity on hypertension and cardiovascular diseases have been well documented (Diaz & Shimbo 2013; Hegde & Solomon 2015; Ma et al. 2021; Verma et al. 2021). The World Health Organization recommends that adults with chronic conditions such as hypertension should do at least 150–300 min of moderate-intensity aerobic physical activity, or at least 75–150 min of vigorous-intensity aerobic physical activity or an equivalent combination of moderate- and vigorous-intensity activity throughout the week. Other key elements of the guidance state that they should also do muscle-strengthening activities at moderate or greater intensity that involve all major muscle groups on two or more days a week and should limit the amount of time spent being sedentary for substantial health benefits (World Health Organization 2020).

Men and women in rural areas of South Africa generally perceived themselves as being highly active (Malambo et al. 2017) because many are involved in moderate-intensity manual labour; however, there is a lack of data on the physical activity of South African adults in rural populations in general and even less knowledge about those who are living with hypertension (Gómez-Olivé et al. 2018). Existing literature (Cook 2007; Gómez-Olivé et al. 2018; Roos et al. 2014) is limited by a single indicator of overall physical activity such as total step count (Roos et al. 2014) and/or self-reported minutes spent in moderate to vigorous physical activity (Gómez-Olivé et al. 2018) without consideration of the types of activities undertaken, which is critical for informing tailored interventions. The coronavirus disease 2019 (COVID-19) pandemic also had an influence on the amount of physical activity participation by individuals because of the limited social contact with the environment (Adebiyi et al. 2021).

In order to implement contextually appropriate physical activity interventions that focus on controlling hypertension, there is a need to better understand the physical activities by sex and work status. Thus, the aim of this study was to provide a profile of the frequency, intensity, duration and domain of self-reported physical activities over a 7-day period in adults with hypertension from a rural sub-district in South Africa.

## Research methods and design

### Study design and participants

The study was a cross-sectional, descriptive population-based study of adults aged 40 years and above with a previous diagnosis of hypertension living in Agincourt rural sub-district in Mpumalanga Province, South Africa. Data were collected during October 2021. Because of COVID-19 regulations at the time of data collection, participants were recruited via telephone, and consent was obtained verbally. Movements and human contact were limited during the time of data collection, and this may influence the level of physical activity reported as well as the use of public transport. Participants were recruited from the Health and Ageing in Africa: A Longitudinal Study of an International Network for the Demographic Evaluation of Populations and Their Health (INDEPTH) community (HAALSI) in South Africa (Gómez-Olivé et al. 2018). The HAALSI study is a cohort of 5059 adults aged 40 years and above living in Agincourt sub-district. This cohort was reported to have a hypertension prevalence of 57% (*n* = 2843) at the time of data collection (Gómez-Olivé et al. 2018).

### Sample size estimation and selection

The inclusion criteria were men and women aged ≥ 40 years, hypertensive (defined by self-reported anti-hypertensive medication use or diagnosed with raised blood pressure of ≥ 140/90mmHg) and living in Agincourt sub-district for at least 6 months prior to the date of data collection. Participants were contacted telephonically using a computer-generated simple random sampling technique. Based on the hypertension prevalence in the study cohort, to achieve a 95% confidence interval with a 5% margin of error, a minimum sample of 339 participants living with hypertension from the HAALSI cohort was required.

### Method of physical activity assessment

The International Physical Activity Questionnaire Long Form (IPAQ-LF) was used to assess the frequency, intensity, duration and domain of physical activities as part of everyday lives (Craig et al. 2003). The IPAQ-LF was initially designed for use with adults up to the age of 69 years old; however, it has also been validated for use in older adults, which indicated that although differences between sub-groups were apparent, the use of the IPAQ-LF is not limited to specific sub-groups (Cleland et al. 2018; Wanner et al. 2016). The IPAQ-LF comprises a set of five activity domains that seek to obtain information on (1) job-related physical activity; (2) transportation physical activity; (3) housework-related physical activity; (4) recreation, sport and leisure time physical activity and (5) time spent sitting. Each domain asks the participant about the intensity of physical activity performed. Vigorous-intensity physical activities were defined as activities that require hard physical effort and make the participant breathe much harder than normal (Hagströmer, Oja & Sjöström 2006). Moderate-intensity physical activities were defined as activities that take moderate physical effort and make the participant breathe somewhat harder than normal (IPAQ Research Committee 2005). The IPAQ-LF considers only those physical activities performed within the last 7 days as well as time spent sitting. Sitting time included time spent sitting while at work, at home, doing course work and during leisure time. This may include time spent sitting at a desk, visiting friends, reading or sitting or lying down to watch television. The frequency is presented as number of days in a week, and the duration is presented as average minutes on a single day for that week. The IPAQ-LF has been shown to have acceptable validity and reliability for the assessment of physical activity among adults of different sex, age and region (Boon et al. 2010; Wanner et al. 2016). A study conducted in 12 countries, over 14 study sites, showed that the IPAQ-LF had excellent one-week test-retest reliability (pooled *r* = 0.81) and acceptable validity (pooled *r* = 0.33) when compared with accelerometer-measured physical activity (Craig et al. 2003). The original version of the IPAQ-LF is available in English (Hagströmer et al. 2006). For our study, data were collected in the participants’ first language (Xitsonga) and were interviewer-administered over the telephone by researchers who are fluent in both English and Xitsonga. The translation process was a three-step back translation method, which involved the translation of the original questionnaire to Xitsonga by an independent translator and then a second translator translated the Xitsonga version back to English. The first author compared the two documents, and any differences were reconciled with the two translators. No adaptations were made to the original version of the questionnaire.

### Statistical analyses

Data were captured on REDCap and analysed using STATA 16. Categorical variables such as sex, work status and age were summarised using frequency and percentage. Because of the uneven distribution of data across physical activity duration and frequency, data were summarised using median and interquartile ranges (IQR). To calculate the differences between the individual types of physical activity from IPAQ-LF between male and female and work status, the Mann-Whitney U test and Krustal-Wallis rank test were used with a level of statistical significance of *p* < 0.05.

### Ethical considerations

This study required clearance from two ethical bodies. The first clearance was obtained from the University of the Witwatersrand Human Research Ethics Committee (HREC – Medical) (clearance number: M 210282). The second clearance was obtained from the local Provincial Department of Health Research and Ethics Committee (clearance number: MP_202106_001). This study was based on the usual ethical principles, such as every person’s right to refuse to participate in the study and to withdraw at any time, as well as respect for all participants and protection of their privacy.

## Results

**FIGURE 1:**
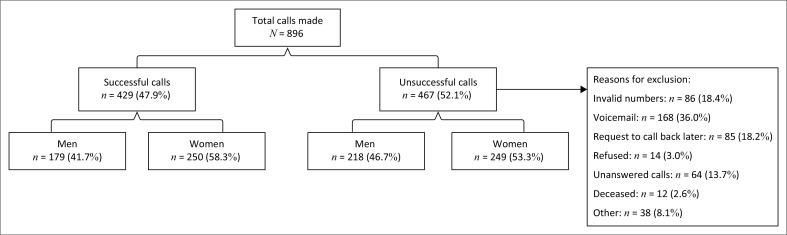
Distribution of included and excluded participants.

A total of 896 calls were made to recruit participants, and 429 complete responses were included in the final analysis (Figure 1). Reasons for exclusion included invalid phone number, voicemail, request to call back later, refused participation, unanswered call, deceased and others. The mean age of included participants was 65.1 years (standard deviation [s.d.] ± 10.9 years), 58% (*n* = 250) of the participants were women and 52% (*n* = 225) had paid or unpaid work (Table 1). Men who had paid or unpaid work were 64.2% while only 44% of women had paid or unpaid work.

**TABLE 1:** Demographic profile of participants.

Variable	Frequency	%	Mean	s.d.
**Age (years)**				
40–49	32	7.5	-	-
50–59	121	28.5	-	-
60–69	126	29.4	-	-
70–79	102	23.8	-	-
80+	48	11.2	65.1	10.9
**Sex**				
Men	179	41.7	-	-
Women	250	58.3	-	-
**Work status**				
Men working	115	64.2	-	-
Men not working	64	35.8	-	-
Women working	110	44.0	-	-
Women not working	140	56.0	-	-

s.d., standard deviation.

### Total physical activity

For all participants, the total weekly physical activity duration was 330 (120–595) min. This consisted of 45 (10–130) min of walking, 120 (45–240) min of moderate activities and 60 (0–180) min of vigorous physical activities.

### Work-related physical activity

Vigorous intensity physical activity duration as part of work was most commonly reported and was higher for men (median = 240 min/day, IQR = 120–390), than women (median= 120 min/day, IQR = 60–240); *p* = 0.003, with men reporting more days in vigorous physical activity (median = 2 days/week, IQR = 0–5) than women (median = 1 day/week, IQR = 0–3); *p* = 0.008. Men also reported more minutes of moderate-intensity activity (median = 120 min/day, IQR = 60–217.5) than women (median = 85 min/day, IQR = 60–180). Men reported walking a median of 40 min/day (IQR = 15–120) over a median of 3 days/week (IQR = 2–7), while women walked a median of 30 min/day (IQR = 15–90) over a median of 3 days/week (IQR = 2–5) (Table 2).

**TABLE 2:** Work-related physical activity (*N* = 225).

PA aspect	Total		Men		Women		*p*
	Median	IQR	Median	IQR	Median	IQR	
Vigorous PA (days per week)	2	0–5	180	120–360	1	0–3	0.002
Vigorous PA (min per day)	180	120–360	2	0–5	120	60–240	0.003
Moderate PA (days per week)	2	0–5	120	60–180	2	0–5	0.475
Moderate PA (min per day)	120	60–180	3	2–5	85	60–180	0.036
Walking (days per week)	3	2–5	30	15–120	3	2–5	0.467
Walking (min per day)	30	15–120	40	15–120	30	15–90	0.353

PA, physical activity; IQR, interquartile range.

### Transportation-related physical activity

Men and women who were working walked longer (median = 30 min/day) than those who were not working (*p* < 0.001). The frequency of walking was also higher for men and women who were working (median = 3 days/week) (*p* = 0.009). No differences were observed in the duration spent travelling in a motor vehicle between men and women (*p* = 0.596); however, working participants travelled in a motor vehicle for more days compared to non-working participants (*p* < 0.001). The duration and frequency of travelling by bicycle were low for all participants (Table 3).

**TABLE 3:** Transportation physical activity (*N* = 429).

Physical activity aspect	Total		Men				Women				*p*
	Median	IQR	Working		Not working		Working		Not working		
			Median	IQR	Median	IQR	Median	IQR	Median	IQR	
Motor vehicle (days/week)	0	0–2	1	0–4	0	0–2	1	0–2	0	0–1	**0.**001
Motor vehicle (mins/day)	60	30–120	60	30–120	60	52.5–150	60	30–120	60	30–120	0.596
Bicycle (days/week)	0	0–0	0	0–0	0	0–0	0	0–0	0	0–0	0.388
Bicycle (mins/day)	25	10–50	40	20–60	30	15–40	0	0–0	7.5	5–10	0.060
Walking (days/week)	2	1–4	3	1–6	2	0–5.5	3	1–3	1	0–2.5	0.009
Walking (mins/day)	30	10–60	30	15–90	25	10–60	30	15–45	15	10–45	0.001

IQR, interquartile range.

### Housework, house maintenance and caring for family

Men and women reported low volumes of vigorous physical activity while doing housework, house maintenance and caring for family (median = 0 days/week). Women, however, reported more days per week performing moderate-intensity physical activities inside the house compared to men (*p* < 0.001). Women also spent more days performing moderate-intensity household activities outside the house compared to men (*p* < 0.001). The overall duration spent on housework, house maintenance and caring for family inside and outside the house was 60 min/day for both men and women (Table 4).

**TABLE 4:** Housework, house maintenance and caring for family (*N* = 429).

Physical activity aspect	Total		Men				Women				*p*
	Median	IQR	Working		Not working		Working		Not working		
			Median	IQR	Median	IQR	Median	IQR	Median	IQR	
Vigorous: heavy lifting, chopping wood or digging in the garden or yard (days/week)	0	0–2	0	0–2	0	0–1	0	0–2	0	0–1	0.018
Vigorous: heavy lifting, chopping wood or digging in the garden or yard (mins/day)	120	60–180	120	60–180	180	120–180	90	60–165	60	60–150	0.052
Moderate: carrying light loads, sweeping, washing windows and raking in the garden or yard (days/week)	2	0–5	0	0–2	0	0–3	3	1–7	3	0–6	0.001
Moderate: carrying light loads, sweeping, washing and raking in the garden or yard (mins/day)	60	45–120	60	35–120	60	30–180	80	60–120	60	45–120	0.555
Moderate: washing windows, scrubbing floors and sweeping inside home (days/week)	2	0–7	0	0–1	0	0–2	5	2–7	5	0–7	0.001
Moderate: washing windows, scrubbing floors and sweeping inside home (mins/day)	60	40–120	60	22.5–105	60	30–180	65	60–120	60	30–120	0.047

IQR, interquartile range.

### Recreation, sport and leisure time physical activity

The overall reported recreation, sport and leisure physical activities were low. No statistical differences by sex and work status were seen. All men and women spent a median of 0 days/week performing recreational, sport and leisure physical activities (Table 5).

**TABLE 5:** Recreation, sport and leisure time physical activity (*N* = 429).

Physical activity aspect	Total		Men				Women				*p*
	Median	IQR	Working		Not working		Working		Not working		
			Median	IQR	Median	IQR	Median	IQR	Median	IQR	
Walking for leisure (days/week)	0.0	0.0–0.0	0.0	0.0–1.0	0.0	0–1	0.0	0–0	0.0	0.0–0.0	0.108
Walking for leisure (min/day)	60.0	30.0–180.0	60.0	45.0–180.0	120.0	30–180	90.0	60–180	52.5	30.0–180.0	0.660
Vigorous leisure: (days/week)	0.0	0.0–0.0	0.0	0.0–0.0	0.0	0–0	0.0	0–0	0.0	0–0	0.948
Vigorous leisure: (min/day)	60.0	30.0–180.0	90.0	60.0–120.0	60.0	30–120	45.0	15–80	60.0	30.0–120.0	0.512
Moderate leisure: (days/week)	0.0	0.0–0.0	0.0	0.0–0.0	0.0	0–0	0.0	0–0	0.0	0.0–0.0	0.140
Moderate leisure: (min/day)	105.0	60.0–120.0	97.5	75.0–142.5	120.0	45–210	60.0	40–120	120.0	120.0–120.0	0.603

IQR, interquartile range.

### Time spent sitting

Men and women who do not work reported spending more time sitting on a weekday (median = 180 min/day) compared to those who work (median = 120 min/day) (*p* = 0.009). No statistical differences were seen for time spent sitting on a weekend (Table 6).

**TABLE 6:** Time spent sitting at work, at home, while doing ‘course’ work and during leisure time (*N* = 429).

Physical activity aspect	Total		Men				Women				*p*
	Median	IQR	Working		Not working		Working		Not working		
			Median	IQR	Median	IQR	Median	IQR	Median	IQR	
Sitting on a weekday (min/day)	120	60–240	120	60–180	180	62–300	120	60–180	180	60–300	**0.009**
Sitting on a weekend (min/day)	150	60–300	150	60–270	180	60–390	150	60–240	180	30–300	0.794

IQR, interquartile range.

## Discussion

This study profiled the frequency, intensity, duration and domain of self-reported physical activities of rural South African adults with hypertension by sex and work status. We found that, overall, participants in our study self-reported meeting the aerobic component of the international guidelines for total physical activity per week when considering physical activities as part of work, transportation, housework, recreation and time spent sitting (Bull et al. 2020).

### Work-related physical activity

Most studies on physical activity for the rural South African population have been conducted on children and adolescents who are not within employment age (Craig, Bland & Reilly 2013; Micklesfield et al. 2014; Sedibe et al. 2014; Van Biljon et al. 2018). We found a higher frequency of physical activity during work (paid or unpaid) for men compared to women, which is in line with the type of employment opportunities found in rural South Africa. Men are usually employed in physical labour jobs such as construction, while women are more likely to be in less active jobs such as self-employment as saleswomen in the formal or informal market (Wilkinson et al. 2017). We also found that the duration of vigorous physical activity at work was higher for men than women, this has face validity as construction work typically involves more time being physically active than selling at the shop or even house-to-house selling (Eveleigh 2023). Although our study did not ask about the type of employment undertaken by the participants, it is evident that sex differences play a role in physical activity as part of work. In our study, employment referred to paid or unpaid work. The most commonly reported unpaid work by other studies in rural South Africa is subsistence farming, which favours men more than women (Eveleigh 2023); hence the higher frequency and duration of physical activity reported by men versus women. Future physical activity interventions for adults with hypertension may consider targeting increased physical activity for employed women in rural settings.

### Transportation and physical activity

Employed men and women in our study spent more days travelling in a motor vehicle. Access to a motor vehicle is associated with driving to the destination as compared to walking and may contribute to enhanced inactivity and/or sedentary (sitting) behaviours (Koohsari et al. 2020; Sugiyama et al. 2020). Although we did not ask participants about ownership of a motor vehicle, it is likely that those who do not own a motor vehicle would rely on walking as a means of transport. Interestingly, employed participants reported more days per week and minutes per day walking as a means of transport compared to their unemployed counterparts. Some possible reasons for higher walking frequency and duration for employed adults could be that their employment does not require or guarantee motor vehicle ownership; therefore, these individuals could spend most time walking to catch public transport for personal, social and family responsibilities compared to unemployed participants who are likely to spend less time using public motor vehicle transport because of cost challenges (Biernat & Piątkowska 2017). Walking as a means of transport is generally higher in rural areas because of multiple livelihood and environmental factors (Shackleton & Hebinck 2018). People walk to fetch water and firewood, to visit friends, to attend social gatherings and to access shops and supermarkets that are usually within walking distance or walk to the taxi rank for farther travel (Collinson 2010; Ragie et al. 2020). There is an opportunity for physical activity interventions in public health to take advantage of the walking opportunities in rural areas to encourage physical activity for hypertensive participants.

### Housework, house maintenance and caring for family

Physical activities such as sweeping, washing, scrubbing floors and raking in the garden at moderate intensities were higher for women compared to men. This is in line with traditional gender roles in African society where women assume more responsibilities in taking care of the family and performing housework and house maintenance inside and outside the house (Oyewumi 2016). Men reported more vigorous physical activity related to housework compared to women, regardless of work status. This finding for housework physical activity reported by men could be linked to gender and sociocultural roles in the household where ‘tougher’ activities in the household are performed by men (Oyewumi 2016). Even though men undertake more vigorous intensity housework and physical activities than women in this setting, their level of hypertension control still remains lower than women (Jardim et al. 2017). This could possibly be because of other men-associated risk factors such as tobacco smoking, alcohol consumption and poor dietary habits (Sulaica et al. 2020).

### Recreation, sport and leisure time physical activity

Physical activity duration and frequency related to recreation, sport and leisure time across both sex and work status were low among our study participants. Rural areas of South Africa lack amenities (Rogerson & Sixaba 2022) for recreation and do not routinely promote recreational physical activity. As seen in this study, hypertensive adults in a rural South African setting spent a reasonable number of days performing housework and paid or unpaid work physical activity tasks, which require physical effort and probably leave less time to devote to leisure activity.

There appears to be an association between physical activity related to leisure and recreation with age (Stodolska 2015), with children, adolescents and youth engaging in more physical activities such as play, swimming and sports than adults (Spencer & Zembani 2011). Other authors (Gómez-Olivé et al. 2017) also reported that younger age and higher education contributed to engaging in walking for leisure. The age of our study population could explain why adults in this study are involved in little to no leisure and recreational physical activities. There is an opportunity to explore what extramural activities adults in a rural setting partake in so that interventions to encourage recreation and leisure physical activity can be implemented. The development of rural areas in South Africa (South African Government 2017) such as building of malls, recreational parks, swimming pools, tarred and paved roads and sidewalks can also be an opportunity to encourage hypertensive adults to participate in physical activity for leisure. Local government should accelerate rural development with a focus on creating recreational opportunities for adults to perform in their leisure time.

### Time spent sitting

Overall self-reported sitting time was slightly higher at weekends than during the week. No known study in rural South Africa has explained this phenomenon. A possible explanation for slightly longer weekend sitting time could be that participants have a relatively high physical activity demand of work, personal and family responsibilities during the week, resulting in people wanting to rest at the weekend. A previous study in rural South Africa (Peltzer & Phaswana-Mafuya 2012) has attributed longer sitting time to obesity, older age, lack of social cohesion and physical limitation. Our study included older adults; we did not assess obesity and participants with physical limitations were excluded. In rural areas, adults typically spend their weekends performing activities that require long sitting times such as attending religious gatherings, community social networks, funerals and other household duties such as washing clothes. Non-working participants reported slightly more sitting time compared to those who work. A report from Biernat & Piątkowska (2017) states that women, smokers and those without full-time employment reported longer sitting time. The phenomenon could not be explained but may be attributed to the fact that employed adults have more responsibilities and less free time than their unemployed counterparts. More information is required regarding the type of activities hypertensive adults perform on weekends for interventions that aim to target physical activity at the weekend.

### Strengths and limitations

This is the first study, to the authors’ knowledge, to provide a physical activity profile of adults with hypertension in Agincourt sub-district. This study provides rich useful data on physical activity participation; however, the study used the IPAQ-LF, which while a validated questionnaire (Boon et al. 2010; Wanner et al. 2016) likely led to error in the measurement associated with inaccurate recall and/or self-report bias (Cleland et al. 2018). Furthermore, we collected data from 32 villages without clustering, which has a potential for under- and over-representation from particular villages. Our study also had limited socio-demographic data, such as indicators of socio-economic status, which would have provided more insight into determinants of physical activity. We collected data during the COVID-19 pandemic where movements and human contact were limited. It is possible that the reported physical activity levels might have been influenced by limited movements including the use of public transport during the pandemic. Majority of our study participants are aged 60 years and older, which is considered retirement age; therefore, future studies could consider providing physical activity profiling by age.

## Conclusion

The findings of this study highlight the influence of sex and gender roles, work status, and environmental and social factors as determinants of physical activity in a hypertensive rural South African adult population. The concept of gender roles appears to influence the intensity and type of physical activities adults perform in a rural South African setting, particularly regarding work-related and housework-related physical activity. Social influences must also be considered; social expectations tend to be internalised by adult men and women, thus adding to the perception that men must perform more strenuous activities than women. This study necessitates a further exploration into the socio-economic factors influencing physical activity for hypertensive adults in a rural setting so that appropriate contextual interventions can be developed and evaluated.
